# Epididymal and ejaculated sperm differ on their response to the cryopreservation and capacitation processes in mouflon (*Ovis musimon*)

**DOI:** 10.1038/s41598-019-52057-0

**Published:** 2019-10-30

**Authors:** Lucía Martínez-Fresneda, Cristina Castaño, Paula Bóveda, Dawit Tesfaye, Karl Schellander, Julián Santiago-Moreno, Francisco A. García-Vázquez

**Affiliations:** 1 Department of Animal Reproduction, Spanish National Institute for Agricultural and Food Research and Technology (INIA), Avda Puerta de Hierro km 5.9, 28040 Madrid, Spain; 2 Department of Physiology, Faculty of Veterinary Science, International Excellence Campus for Higher Education and Research “Campus Mare Nostrum”, University of Murcia, Campus de Espinardo, 30100 Murcia, Spain; 3 Department of Animal Breeding and Husbandry, Institute of Animal Science, University of Bonn, Endenicher Allee 15, 53115 Bonn, Germany; 4 Biomedical Research Institute of Murcia, IMIB-Arrixaca, Crta. Buenavista s/n, El Palmar, 30120 Murcia, Spain

**Keywords:** Animal biotechnology, Zoology

## Abstract

Spermatozoa must undergo the process of capacitation to fertilize the egg which involves a cell destabilizing process. Capacitation-like changes such as protein tyrosine phosphorylation (PTP) are associated with cryopreservation. The aim of this study was to compare the cryoresistance and capacitation response of epididymal and ejaculated sperm of European mouflon (*Ovis musimon*). Post-thaw sperm parameters were analysed from epididymal and ejaculated samples cryopreserved by slow-freezing or ultrarapid-freezing for comparison. Sperm capacitation status was assessed by the semiquantification of PTP levels, cell localization of PTP and kinematic clustering. Epididymal sperm had higher cryoresistance than ejaculated sperm in both freezing techniques, and slow-freezing rendered better results than ultrarapid-freezing in both sperm samples. Ejaculated sperm had higher PTP levels than epididymal sperm and, additionally, ejaculated sperm showed higher phosphorylation in capacitating (CA) than in non-capacitating (NCA) conditions while there was no effect of medium in epididymal sperm. There was a higher tail PTP in CA than in NCA conditions in both types of sperm. Kinematic analysis revealed that the cluster associated with hyperactivated movement increased in ejaculated sperm incubated in CA whereas no effect of medium was observed in epididymal sperm clusters. In conclusion, epididymal sperm showed better freezability and lower capacitation status compared to ejaculated sperm.

## Introduction

The European mouflon (*Ovis musimon*), a wild ancestor of domestic sheep, contributes to the Mediterranean ecosystem biodiversity and is highly sought in the hunting industry. Important habitat fragmentation due to increased human activity in the environment has led wild ruminants such as mouflon, to inbreeding problems. Assisted reproductive techniques (ART) such as artificial insemination using frozen-thawed sperm, beyond its important role in domestic livestock breeding programs, support animal conservation and help maintain genetic diversity in domestic and wild species^1,2^. In addition to ejaculated sperm, collection and cryopreservation of epididymal samples from genetically valuable dead individuals is a good source of genetic material to be preserved in germplasm banks. The development of efficient sperm freezing techniques are especially important when working with wild species, and ultrarapid-freezing protocols for epididymal and ejaculated sperm cryopreservation have been recently reported^3–6^. Previous studies that optimized freezing protocols in mouflon sperm recommended the use of sucrose and glycerol as cryoprotectants during ultrarapid and slow-freezing respectively^3^. Nevertheless, it has been shown that the handling and storage affect sperm quality parameters and, in some cases, fertility^7^.

The composition of epididymal sperm is remodeled during ejaculation, thus the proteome of ejaculated sperm is different from that of epididymal sperm in ram^8^ and boar^9^. Additionally, variations in the composition of lipids has been reported for ram sperm, with testicular sperm containing higher concentrations of phospholipids and cholesterol than ejaculated sperm^10^. Moreover, the medium surrounding sperm cells differs between both types of samples and, as epididymal fluid does not contain secretions of the accessory glands, and seminal plasma does. These differences between seminal and epididymal fluid have been reported to affect freezing resistance in different species. Ram epididymal sperm was found to be more resistant than ejaculated sperm to osmotic stress, cold shock and cryoprotective agents exposure^11,12^. Similarly, epididymal sperm from bulls^13^, boars^14^ and stallions^15^ are more resistant to the cryopreservation process than ejaculated sperm.

Besides advancements in sperm cryopreservation, further studies are needed to evaluate the process of capacitation during *in vivo* or *in vitro* fertilization procedures (before being able to fertilize spermatozoa undergo a series of modifications known as capacitation process^16,17^). During sperm storage in the cauda of the epididymis the luminal fluid microenvironment prevents the destabilizing processes associated with sperm capacitation^18–20^. Upon ejaculation, sperm cells undergo a series of modifications that entail sperm capacitation, a process modulated by seminal plasma^21,22^ and female reproductive tract fluids^23,24^. Thus, sperm capacitation has been described as a series of physiological changes in both plasma membrane and intracellular components that allow sperm cells to undergo the acrosome reaction and fertilize the egg^25^. Although sperm cells require a period of time in the female genital tract in order to acquire the fertilizing capacity^26,27^, sperm capacitation can be also accomplished *in vitro*.

The cascade of molecular events associated with sperm capacitation *in vitro* starts with removal of cholesterol from sperm plasma membrane by cholesterol acceptors (e.g. serum albumin) and channel activation to induce the influx of HCO_3_^−^ and Ca^2+^. The resulting increases in intracellular pH and membrane hyperpolarization activate adenylyl cyclase (AC), which increases intracellular cyclic adenosine monophosphate (cAMP) levels and protein kinase A (PKA) activation. PKA stimulates the activation of kinases and/or the inhibition of phosphatases which leads to an increase of protein tyrosine phosphorylation (PTP)^28^. Thus, sperm PTP levels are a marker of sperm capacitation status in species such as ram^29^, bull^30^, mouse^28^, human^31^, boar^32^ and stallion^33^. Moreover, the sperm PTP pattern is different between epididymal and ejaculated bull sperm^34^, but, to our knowledge, a pattern has yet to be described in the ram.

Modifications during sperm capacitation include changes of membrane properties, intracellular constituents, enzymatic activity and motility pattern^35^. Motility activation is a very early event in sperm capacitation which is followed by the slower event of hyperactivated motility^16^. Hyperactivation was first described by Yanagimachi^36,37^ as a vigorous movement characterized by asymmetrical and high-amplitude flagellar beats that sperm cells acquire before fertilization. Nevertheless the association between capacitation and hyperactivated motility is not yet clear since divergent pathways have been suggested for each event^38,39^. Sperm samples contain a heterogeneous population of cells with different physiological and structural characteristics. Changes in the milieu during sperm capacitation, such as bicarbonate levels, affect each spermatozoa differently^40^, thus the identification of sperm clusters based on kinematic parameters can be a valuable tool to distinguish hyperactivated patterns of motility^41,42^. The increase of curvilinear velocity (VCL) and amplitude of lateral head displacement (ALH) accompanied by the decrease of linearity (LIN) have been associated with ram sperm hyperactivation and capacitation-related changes^43–45^.

Due to the different composition and physiological status in epididymal and ejaculated sperm, we hypothesized that freezing resistance and capacitation response differ between both types of sperm samples. The objectives were (i) to compare sperm freezability of epididymal and ejaculated sperm using slow-freezing and ultrarapid-freezing techniques and (ii) to compare the capacitation response of frozen-thawed epididymal and ejaculated sperm samples of European mouflon (*Ovis musimon*).

## Results

### Experiment 1: Effect of sperm source (epididymal or ejaculated) on freezability (slow or ultrarapid)

Prior to the freezing process, fresh sperm motility and acrosome integrity (AI) did not differ between epididymal and ejaculated samples (73.1 ± 4.6% *vs* 66.6 ± 2.9% and 86.7 ± 2.1% *vs* 88.6 ± 2.0%, respectively). Membrane integrity (MI) was higher in fresh epididymal sperm than in fresh ejaculated sperm by eosin-nigrosin staining (EN) (86.1 ± 1.8% *vs* 71.5 ± 3.4%; p < 0.001) and by the hypo-osmotic swelling test (HOST) (86.7 ± 3.0% *vs* 68.7 ± 3.6%; p < 0.001).

**Figure 1:**
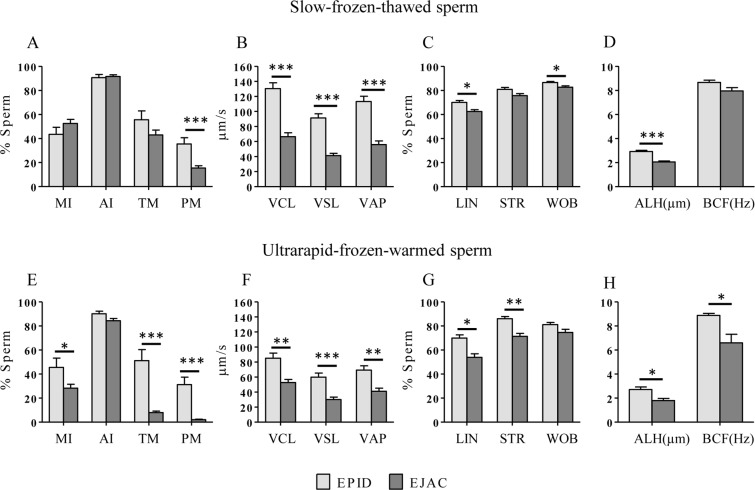
Mouflon sperm quality parameters of thawed/warmed epididymal (n = 12; light grey bars) and ejaculated (n = 25; dark grey bars) sperm after slow-freezing-thawing (**A**–**D**) and ultrarapid-freezing-warming (**E–H**). Data are expressed as mean ± s.e.m. and asterisks indicate significant differences between epididymal and ejaculated sperm (*p < 0.05; **p < 0.001; ***p < 0.0001). MI: membrane integrity; AI: acrosome integrity; TM: total motility; PM: progressive motility; VCL: curvilinear velocity; VSL: straight-line velocity; VAP: average path velocity; LIN: linearity; STR: straightness; WOB: wobble; ALH: amplitude of lateral head displacement; BCF: beat-cross frequency.

Overall, after the thawing/warming process epididymal sperm showed higher quality parameters than ejaculated sperm (Fig. 1). Using the slow-freezing technique, the post-thaw progressive motility (PM), VCL, straight-line velocity (VSL), average path velocity (VAP), ALH (p < 0.0001), LIN and wobble (WOB) (p < 0.05) were higher in epididymal than ejaculated sperm (Fig. 1A–D). Using the ultrarapid-freezing technique, total motility (TM), PM, VSL (p < 0.0001), VCL, VAP, straightness (STR) (p < 0.001), MI, LIN, ALH and beat-cross frequency (BCF) (p < 0.05) were higher in epididymal than ejaculated sperm (Fig. 1E–H).

Comparing both freezing techniques, sperm quality parameters after the thawing/warming process were higher using the slow-freezing than the ultrarapid-freezing (Fig. 1). Frozen-thawed epididymal sperm showed higher VCL, VSL, VAP (p < 0.001) and WOB (p < 0.05) than ultrarapid-frozen-warmed epididymal samples. Frozen-thawed ejaculated sperm showed higher MI, TM, PM (p < 0.0001), AI, VCL, VSL, VAP, LIN and WOB (p < 0.05) than ultrarapid-frozen-warmed ejaculated sperm. Based on these results, samples cryopreserved by slow-freezing were used in the experiment 2.

### Experiment 2: Effect of sperm source (epididymal or ejaculated) on capacitation status

#### Evaluation of sperm PTP by immunoblotting

**Figure 2:**
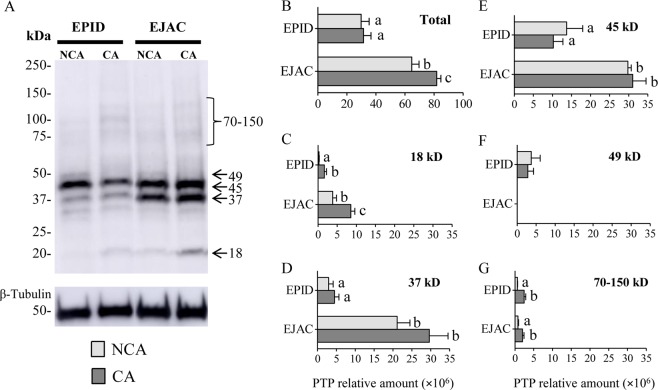
Immunoblotting analysis of the protein tyrosine phosphorylation (PTP) in epididymal (EPID; n = 4) and ejaculated (EJAC; n = 5) mouflon sperm incubated in non-capacitating (NCA: light grey bars) and capacitating (CA: dark grey bars) medium (β-tubulin was used as loading control) (**A**). Total semiquantification of lanes is shown in graph B and semiquantification corresponding to 18 kD, 37 kD, 45 kD, 49 kD, and 70–150 kD molecular weight bands is shown in graphs (**C**–**G**). Data are expressed as mean ± s.e.m. Different letters (a, b, c) in bar graphs indicate significant differences between groups of study (p < 0.05).

Immunoblotting results (Fig. 2A) showed a higher degree of PTP signal in ejaculated sperm incubated in capacitating (CA) than in non-capacitating (NCA) conditions (p < 0.05; Fig. 2B) while no effect of incubation medium on the total PTP lane semiquantification was found in epididymal sperm. When comparing both types of samples, the PTP signal was higher in ejaculated than epididymal sperm (p < 0.05; Fig. 2B).

The PTP signal of specific protein bands was affected by incubation medium as well as by sperm source (Fig. 2C–G). The 18 kDa protein band showed higher PTP signal in CA than in NCA in both epididymal and ejaculated samples (p < 0.05; Fig. 2C). This band showed higher signal in ejaculated than epididymal sperm under CA conditions (p < 0.05). The 37 kDa and 45 kDa protein bands were not affected by incubation medium but had higher PTP signal in ejaculated than epididymal sperm (p < 0.05; Fig. 2D,E). The PTP signal of the 49 kDa protein band was not affected by incubation medium and was detected in epididymal but not in ejaculated samples (Fig. 2F). The band region 70–150 kDa showed higher PTP signal in CA than in NCA in both epididymal and ejaculated samples (p < 0.05; Fig. 2G).

#### Immunolocalization of sperm PTP

**Figure 3:**
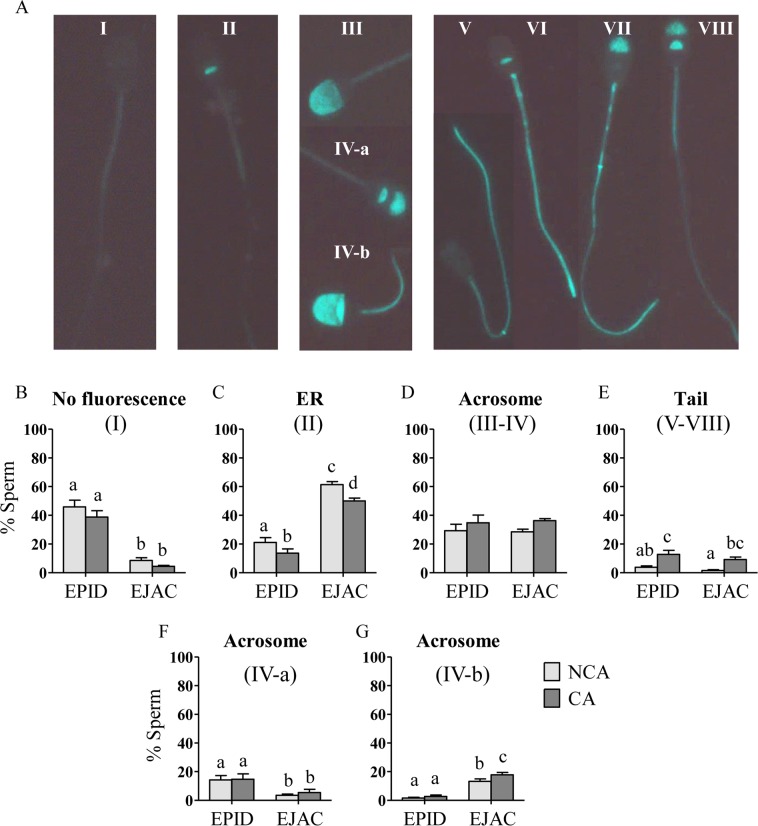
Subpopulations of epididymal (EPID; n = 5) and ejaculated (EJAC; n = 5) sperm incubated in non-capacitating (NCA: light grey bars) and capacitating (CA: dark grey bars) medium according to the location of phosphorylated proteins: no fluorescence (pattern I; **A**,**B**), ER fluorescence (pattern II; **A**,**C**), acrosome fluorescence (patterns III-IV; **A**,**D**) and tail fluorescence (patterns V-VIII; **A**,**E**). Results of acrosome fluorescence patterns IV-a and IV-b are shown in graphs (**F**,**G**). Different letters (a, b, c, d) in bar graphs indicate significant differences between groups of study (p < 0.05).

Representative images of sperm PTP fluorescence patterns are shown in Fig. 3A. Average values of each immunofluorescence pattern during the 3 h incubation are shown in Fig. 3B–G. Incubation medium did not affect the percentage of sperm showing no fluorescence (pattern I) or the percentage of sperm showing acrosome fluorescence (pattern III-IV) of both epididymal and ejaculated sperm (Fig. 3B,D). Interestingly, the percentage of sperm showing equatorial region (ER) fluorescence (pattern II) was lower in CA than in NCA and sperm showing tail fluorescence (patterns V-VIII) was higher in CA than in NCA in both epididymal and ejaculated sperm (p < 0.05; Fig. 3C,E).

An effect of sperm source was found in the PTP immunolocalization with a higher percentage showing no fluorescence (pattern I) in epididymal than ejaculated sperm (p < 0.0001; Fig. 3B) and, inversely, a higher percentage showing ER fluorescence (pattern II) in ejaculated than epididymal sperm (p < 0.0001; Fig. 3C). No difference was found between epididymal and ejaculated sperm regarding acrosome and tail fluorescence (patterns III-VIII; Fig. 3D,E). Regarding the different patterns of acrosome fluorescence, the percentage of sperm showing pattern IV-a was higher in epididymal than in ejaculated sperm (p < 0.05; Fig. 3F). In addition, pattern IV-b was more frequent in ejaculated than epididymal sperm (p < 0.0001; Fig. 3G) and higher in ejaculated sperm incubated in CA than in NCA (p < 0.05; Fig. 3G). Ejaculated sperm showed a time-dependent decrease of acrosome fluorescence pattern IV-b from 0 to 3 h in both media (NCA: 21.2 ± 3.6% *vs* 5.4 ± 1.4%; CA: 25.6 ± 3.9% *vs* 13.4 ± 1.0%; p < 0.0001).

A time-dependent increase from 0 to 3 h was found in ejaculated sperm tail fluorescence incubated in CA (2.0 ± 2.0% *vs* 14.8 ± 3.8%; p < 0.05) while no time effect was found in NCA (1.0 ± 1.0% *vs* 2.6 ± 1.7%). No effect of time from 0 to 3 h was found on epididymal sperm tail fluorescence (CA: 8.8 ± 4.4% *vs* 10.6 ± 5.6%; NCA: 3.0 ± 1.8% *vs* 3.0 ± 1.4%), and there was no interaction between treatment and time in any evaluated parameter.

#### Sperm motility clusters during sperm capacitation

**Figure 4:**
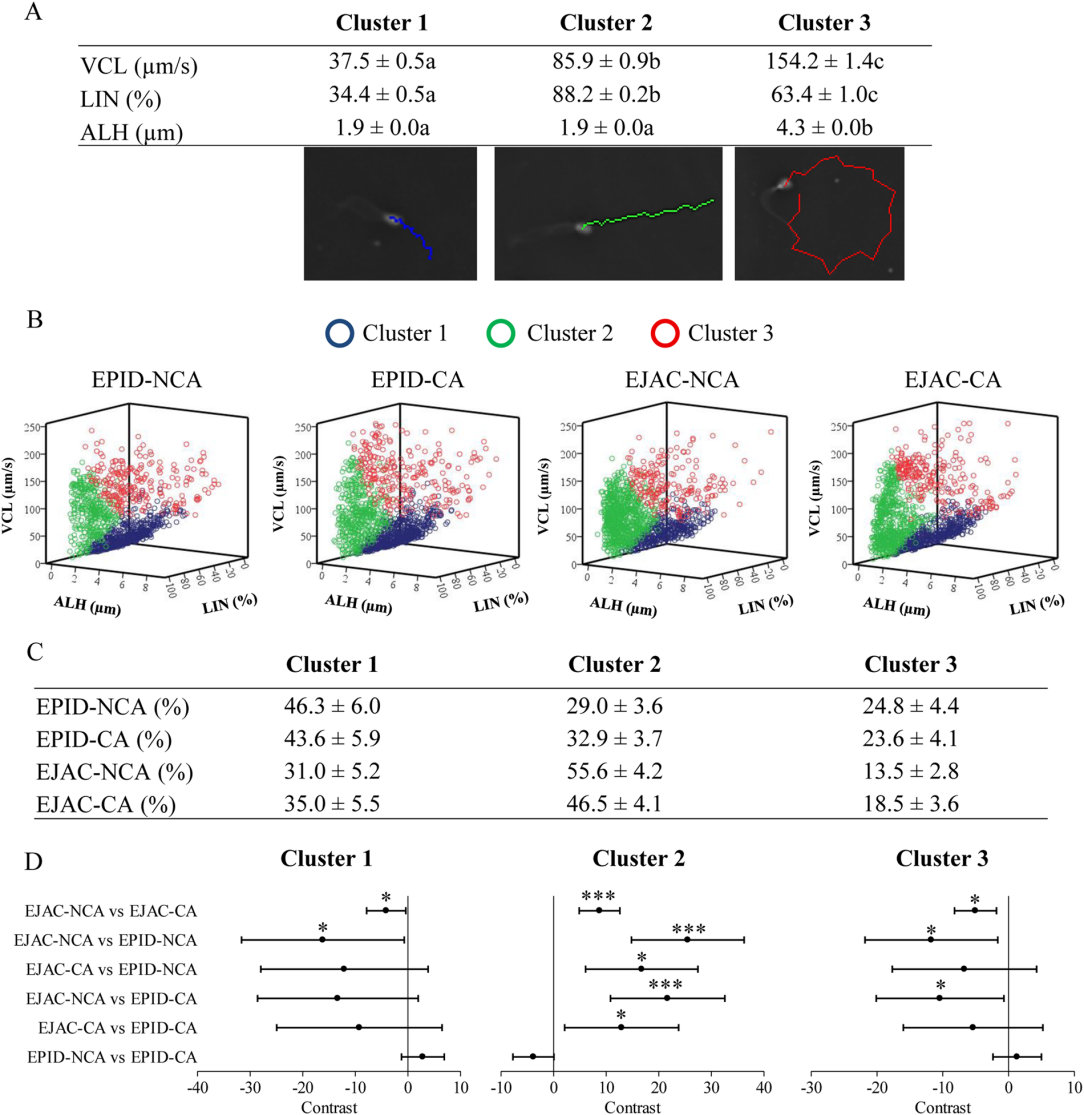
Clustering analysis of epididymal (EPID) and ejaculated (EJAC) sperm trajectory incubated in non-capacitating (NCA) and capacitating (CA) medium. (**A**) Clusters were defined using the kinetic parameters VCL (curvilinear velocity), LIN (linearity) and ALH (amplitude of lateral head displacement). Cluster 1: sperm cells with the lowest kinetic parameters (representative sperm blue trajectory in the image); Cluster 2: sperm with the most linear trajectory (representative sperm green trajectory in the image); Cluster 3: sperm with the fastest and most curvilinear trajectory (representative sperm red trajectory in the image). Different letters within a row indicate significant differences (p < 0.0001). (**B**) Scatter plots show the correlation between clusters of each group. (**C**) Percentage of sperm belonging to each cluster (mean ± s.e.m.). (**D**) Contrast graphs show the differences between percentages of sperm of each group to analyze the probability that they belong to each cluster, therefore values either larger than 0 or smaller than 0 express significant difference between groups (95% confidence interval of differences; *p < 0.05; ***p < 0.0001).

Three sperm clusters were identified with the following characteristics: Cluster 1 consisted of sperm with slow non-linear movement (lowest VCL, LIN and ALH), cluster 2 consisted of sperm with the most linear trajectory (medium VCL, highest LIN and low ALH) and cluster 3 included sperm with the fastest and most curvilinear trajectory (highest VCL, medium LIN and highest ALH). Based on these motility characteristics, cluster 1 and cluster 2 were associated with a non-hyperactivated status whereas cluster 3 was associated with a hyperactivated status. Clusters’ kinetic parameters are shown in Fig. 4A,B.

The percentages of sperm of each experimental group and each cluster are shown in Fig. 4C and the difference between groups (contrasts) are shown in Fig. 4D. Regarding the effect of incubation media, epididymal sperm showed no difference between NCA and CA conditions in all clusters (Fig. 4C,D): cluster 1 (46.3 ± 6.0% *vs* 43.6 ± 5.9%), cluster 2 (29.0 ± 3.6% *vs* 32.9 ± 3.7%) and cluster 3 (24.8 ± 4.4% *vs* 23.6 ± 4.1%). Conversely, clusters of ejaculated sperm were affected by incubation media: ejaculated sperm incubated in NCA had lower proportion of sperm in cluster 1 (31.0 ± 5.2% *vs* 35.0 ± 5.5%; p < 0.05), higher proportion of sperm in cluster 2 (55.6 ± 4.2 *vs* 46.5 ± 4.1%; p < 0.0001) and lower proportion of sperm in cluster 3 (13.5 ± 2.8% *vs* 18.5 ± 3.6%; p < 0.05) than sperm incubated in CA. Differences regarding sperm source were found in all clusters (Fig. 4C,D): the probability to belong to cluster 1 was lower in ejaculated than in epididymal sperm in NCA conditions (p < 0.05); the probability to belong to cluster 2 was higher in ejaculated than in epididymal sperm in CA (p < 0.05) and NCA (p < 0.0001) conditions; the probability to belong to cluster 3 was lower in ejaculated than in epididymal sperm in NCA conditions (p < 0.05).

An interaction between time, media and sperm source was found in cluster 3 (p < 0.0001) whereas no time interaction was found in clusters 1 and 2. Ejaculated sperm cells of cluster 3 decreased from 0 to 3 h in both CA (21.2 ± 4.2% *vs* 11.6 ± 2.9; p < 0.05) and NCA (20.4 ± 4.1% *vs* 9.2 ± 2.5%; p < 0.0001). Epididymal sperm of cluster 3 decreased from 0 to 3 h only in CA conditions (31.0 ± 5.2% *vs* 21.4 ± 4.6%; p < 0.05) whereas no time interaction was found in NCA conditions (from 22.8 ± 4.6% *vs* 25.9 ± 5.1%).

## Discussion

The growing interest in ART has led to new investigations on sperm cryopreservation and sperm functionality in wild species. In the present work, the effect of sperm source (epididymal *vs* ejaculated) on the sperm freezability and the capacitation response were evaluated. Briefly, both types of sperm showed higher cryoresistance during slow- rather than ultrarapid-freezing, cryosurvival was improved in epididymal sperm and capacitation status was better in ejaculated sperm, thus the initial hypothesis is accepted.

Mouflon sperm was more sensitive to ultrarapid-freezing than slow-freezing as reported in a previous study^3^. Nevertheless, ultrarapid-freezing rendered acceptable post-thaw sperm quality parameters and is deemed, convenient when a faster and simpler technique is required in field conditions. In addition, the ultrarapid-freezing technique had a higher detrimental effect in ejaculated than in epididymal sperm which is in agreement with the higher resistance of epididymal samples during its processing reported in other species^11,13–15,46^. The different cryotolerance between epididymal and ejaculated sperm could be related to differences of plasma membrane composition. It has been suggested that the susceptibility to the freezing process is higher in ejaculated than epididymal ram sperm due to lower phospholipid and cholesterol content^10,47^ because high levels of cholesterol content increase sperm resistance to cold-shock and freezing^48,49^. Although mouflons show a more restrictive period of sexual activity compared to domestic rams, both species show seasonal fluctuations of the reproductive hormones and testicular diameter^50^. Furthermore, both domestic and wild rams produce high quality semen throughout the year with seasonal variations of the sperm freezing resistance^51^. Spermatogenic activity in the testis is higher in the middle than at the end of the rutting season whereas the freezability of ejaculated sperm is higher at the end of the rutting season in domestic and wild rams^51^. It would be interesting to investigate if these changes of freezability that occur during the rutting season are associated with the capacitation status as it happens between epididymal and ejaculated sperm.

Furthermore, mass spectrometry studies have shown that the sperm proteome is remodeled during ejaculation in the ram and the boar^8,9^. This affects sperm cryoresistance since sperm proteome has been associated with sperm freezability in different species such as ram^52^, bull^53^ and boar^14,54^. Further studies are needed to identify which proteins play a major role in sperm freezability in small ruminants.

Seminal plasma and epididymal fluid have different compositions^55^ that can affect the cryoresistance and capacitation status of ejaculated and epididymal sperm, respectively. The role of seminal plasma during semen processing still needs to be clarified^56–59^ since some studies report a beneficial effect of seminal plasma during sperm processing^60–64^ while others report a detrimental effect^65,66^. Nevertheless, it is evident that seminal plasma composition differs between species^58^, individuals^67^ and seasons^68^ which could explain discrepancies between studies. Although seminal plasma was removed before freezing of ejaculated sperm in the present study, the interaction between sperm and seminal plasma components occurs in a short period of time, and some components, such as the binder of sperm proteins (BSPs), bind to sperm upon ejaculation and play a key role during later membrane modifications^69^. These BSPs promote sperm capacitation and have a beneficial role in sperm function but, at the same time, these changes in the sperm membrane increase sperm sensitivity to cooling and are detrimental for sperm storage^56^. The lumen of cauda epididymis provides the optimal microenvironment for sperm storage reservoir^70^, and epididymal fluid is able to maintain sperm cells in a quiescent status during long periods of time. Although some specific components of seminal plasma are able to prevent and repair the cold-shock damage to sperm^57,71^ many of the proteins present in seminal plasma have been negatively correlated with sperm preservation ability^72^. In the present study, epididymal sperm showed higher post-thaw quality and lower PTP than ejaculated sperm. The study of the protein and lipid composition of epididymal and ejaculated sperm could elucidate specific components to use as supplements in freezing extenders to increase the sperm cryoresistance. Moreover, comparative studies between the epididymal fluid and seminal plasma composition could improve freezing and capacitation medias used in ARTs. It is suggested that ejaculated sperm show lower cryoresistance than epididymal sperm due to the capacitation-like changes induced by seminal plasma upon ejaculation. Seminal plasma contains factors that prevent and/or facilitate capacitation-like changes^22,73,74^ thus it is unclear how it impacts sperm capacitation^58^. Some studies reported a decrease of sperm PTP after incubation with seminal plasma^29,75^ while others reported an increase of sperm capacitation-like changes after incubation with seminal plasma proteins^21^. What has been demonstrated is that seminal plasma enhances ejaculated ram sperm transit through the cervix of the ewe while epididymal sperm transit is compromised^76^. This supports our findings regarding the higher PTP levels found in ejaculated when compared to epididymal sperm, which suggests a higher level of capacitating factors in seminal plasma than in epididymal fluid.

In the present study, the PTP pattern differed between capacitating and non-capacitating conditions and between epididymal and ejaculated sperm. The phosphorylation of the 18 kDa band and the 70–150 kDa band region was higher under capacitating conditions than under non-capacitating conditions in both types of sperm. The phosphorylation pattern of mouflon sperm shows high similarity to the band pattern reported by Grasa *et al*.^77^ in domestic ram sperm, thus these bands could be used as capacitation markers. Regarding the differences between both types of sperm, the 18 kDa and 37 kDa protein bands were more phosphorylated in ejaculated than in epididymal sperm. In accordance with these results, Pini *et al*.^74^ reported a time dependent increase of the 18 kDa band phosphorylation and a higher phosphorylation of the 37 kDa band in ram epididymal sperm incubated with BSP 5, an abundant seminal plasma protein in this species. The 45 kDa protein band signal was also higher in ejaculated than in epididymal mouflon sperm in accordance with Pérez-Pé *et al*.^29^ who reported phosphorylation of the 45 kDa band in ram sperm.

Regarding the PTP immunolocalization, incubation under capacitating conditions increased the tail phosphorylation in both epididymal and ejaculated sperm. A time-dependent increase of tail phosphorylation was found in ejaculated sperm incubated under capacitating conditions^78,79^ while no effect of time was found in epididymal sperm tail phosphorylation. A positive correlation between flagellum PTP and sperm ZP-binding ability^80^ may be associated with the faster ova penetration that ejaculated sperm show compared to epididymal sperm^81,82^ and with the time dependent increase of ejaculated sperm tail phosphorylation found in the present study. Beside these differences, ram sperm capacitation can be accomplished in ejaculated and cauda epididymal sperm, showing similar fertilizing ability in studies performed *in vivo* and *in vitro*^83,84^ for both types of sperm.

The observation that the percentage of cells showing ER fluorescence was higher in NCA medium correlates with previous work in ram sperm capacitation, which reported a predominance of tyrosine phosphoproteins in the equatorial segment under non-capacitating conditions^78^. Although no effect of medium was found on acrosome phosphorylation, it is noteworthy that epididymal and ejaculated sperm showed different pattern of acrosome phosphorylation. Epididymal sperm showed phosphorylation mostly in the apical region of the acrosome (pattern IV-a) while ejaculated sperm showed phosphorylation in the whole acrosome (pattern IV-b) which suggests differences on the phosphorylated acrosomal proteins. Head PTP has been associated with capacitation^85^ which supports the higher PTP quantification observed in ejaculated sperm.

Mouflon sperm clusters described here are in accordance with those described in domestic ram^86^ and boar sperm^87^ during capacitation. Our results show that kinematic parameters followed an hyperactivated pattern in ejaculated sperm under capacitating conditions but not in epididymal sperm which supports the immunoblot and IFF results. Regarding changes of sperm subpopulations during incubation, García-Álvarez *et al*.^86^ found a time dependent increase of hyperactivated sperm subpopulation whereas Gimeno-Martos *et al*.^88^ reported a decrease of hyperactivated sperm subpopulation during the incubation time, in accordance with our results. We can speculate that the time-dependent decreased found only in cluster 3 could be related with a faster loss of sperm viability once they are capacitated, thus cells belonging to this cluster change to an immotile status during the incubation procedure. The presence of capacitated-like cells in the NCA condition is probably related with capacitation-like changes induced by the cryopreservation process^89,90^ explained by the fact that frozen-thawed sperm was used for the present sperm capacitation study.

## Conclusions

In conclusion, both the slow- and ultrarapid-freezing techniques are suitable for epididymal sperm cryopreservation, whereas post-thaw quality of ejaculated sperm is markedly affected by the ultrarapid-freezing in mouflon. Ejaculated sperm showed lower freezability but higher level of response to the capacitation process than epididymal sperm.

## Methods

All chemicals were purchased from Sigma-Aldrich (Madrid, Spain) unless otherwise noted.

### Ethics

Procedures were approved by the INIA Ethics Committee following the European Union Directive 2010/63/UE.

### Animals and sample collection

All animals included in the study were 3 to 7 years old. Samples were collected in January to avoid changes of sperm properties associated with the season. Epididymides were collected from testicles of animals legally culled at the Game Reserve of Cazorla (37°N latitude, Jaen, Spain). Epididymal sperm were collected within 8 h after death based on previous studies that evaluated the effect of length post-mortem collection on sperm quality^91–93^. Epididymal sperm samples were collected by retrograde flushing using a cannula to flush 1 mL of the freezing extender from the ductus deferens to the cauda of the epididymis as previously described^94^. Diluted sperm samples were collected in a petri dish after making a cut at the end of the cauda.

Ejaculated sperm samples were collected from anesthetized mouflons located at the Animal Reproduction Department of the Spanish National Institute for Agricultural and Food Research and Technology (INIA, 40°N latitude, Madrid, Spain) and at the Córdoba Zoological Garden (37°N latitude, Córdoba, Spain). Animals were maintained under natural day length conditions. Ejaculates were collected by the transrectal ultrasound guided massage technique (TUMASG) described by Santiago-Moreno^95^. During this procedure, animals were anesthetized using a combination of intravenous ketamine hydrochloride (0.5 mg/kg; Imalgene-1000; Rhône Mérieux), detomidine (50 µg/kg; Domosedan; Pfizer Inc.) and tiletamine-zolazepan (0.5 mg/kg; Zoletil-100; Virbac España SA). Animals were maintained with isofluorane (Isobavet; Intervet Schering-Plough Animal Health) and anaesthesia was reversed with yohimbine hydrochloride (0.7 mg/kg: half intravenous and half intramuscular; Sigma, Zwijndrecht, The Netherlands).

### Sperm cryopreservation

Slow-freezing and ultrarapid-freezing methods were used as previously described in other studies^3,96^. Briefly, ejaculates were diluted (1:1) in TCG (Tris 313.7 mM, Citric acid 104.7 mM, Glucose 30.3 mM) and centrifuged (900 × *g*, 20 min) to discard the seminal plasma whereas epididymal fluid was not removed prior to the freezing process. Ejaculated sperm was diluted with the freezing extender to a final concentration of 100 × 10^6^ sperm/mL while epididymal sperm was diluted to 800 × 10^6^ sperm/mL. The freezing extender contained Tris (95.8 mM), TES (210.6 mM), glucose (10.1 mM), 0.08% (w/v) streptomycin, 0.08% (w/v) penicillin, 6% (v/v) clarified egg yolk and either 5% (v/v) glycerol for the slow-freezing or 100 mM sucrose for the ultrarapid-freezing. Conventional slow-freezing in straws implied equilibration at 5 °C for 180 min in ejaculated sperm and for 75 min in epididymal sperm. Samples were loaded in 0.25 mL straws that were exposed to liquid nitrogen (LN) vapor using a metal rack that held the straws in a styrofoam box 5 cm above the LN surface for the last 10 min before being immerse in LN. In the ultrarapid-freezing both types of samples implied only 30 min equilibration at 5 °C and 50 µl sperm pellets were directly plugged into LN using a pipette and were stored in vials. Samples were stored in LN tanks between 4 and 12 months before being thawed/warmed. Straws were thawed in a water bath at 37 °C for 30 s while sperm pellets were warmed at 60–70 °C for ~3 s using a thermoregulated metal plate with conical shape (DDP-70, INIA, Madrid, Spain). Thawed/warmed sperm samples were submitted to a density gradient centrifugation technique by BoviPure (Nidacon International AB, Gothenburg, Sweden) in order to discard the dead/immotile cells as previously described^97^. BoviPure was diluted with BoviDilute (Nidacon International AB, Gothenburg, Sweden) to concentrations of 80% and 40% (v/v) to obtain the bottom layer medium and the top layer medium respectively. Density gradient columns were prepared in 15 mL Falcon tubes by adding equal volumes of bottom and top layers. The total volume of the column was 1 mL per 333 × 10^6^ sperm. Frozen-thawed sperm were gently placed on the top of the column and centrifuged at 300 × *g* for 20 min. The pellet was recovered to evaluate the sperm quality parameters.

### Sperm quality assessment

Slides, coverslips, semen extender and microscope plate were warmed to 37 °C prior to motility evaluation. Subjective motility was assessed placing 5 µl sperm sample in a slide covered with a coverslip (18 × 18 mm). Motility was calculated as the average of 5 different fields evaluated in the center of the coverslip. Samples were diluted in the same medium used for each experiment to evaluate the motility in accordance with the experimental conditions. In experiment 1, sperm motility parameters were assessed by Sperm Class Analyzer v.4.0. software (SCA CASA-mot, Microptic S.L., Barcelona, Spain) equipped with a Nikon microscope (Eclipse 50i, Tokyo, Japan). Sperm was diluted in the freezing extender and 3 µl drops were placed in a Leja eight-chamber slide (Leja Products B.V., Nieuw Vennep, The Netherlands). In experiment 2, motility parameters were evaluated using the motility module of ISAS (PROiSER R + D S.L., Valencia, Spain) equipped with a Nikon microscope (Eclipse E200, Tokyo, Japan). In this case, sperm samples were diluted in TALP medium [100 mM NaCl, 3.1 mM KCl, 0.4 mM MgCl_2_, 21.6 mM Na lactate, 2 mM CaCl_2_, 0.3 mM NaH_2_PO4, 5 mM Glucose, 100 mM Hepes, 1 mM Na pyruvate (pH 7.3 and osmotic pressure 295–305 mOsm/L)]. A 4 µl drop was placed in a Spermtrack Chamber (20 µm, PROiSER R + D S.L., Valencia, Spain). In both experiments, a minimum of three fields and 500 sperm tracks per sample were captured with the 10 × negative-Ph1 objective and the following kinematic parameters were evaluated: TM (%), PM (%), VCL (µm/s), VSL (µm/s), VAP (µm/s), LIN (%), STR (%), WOB (%), ALH (µm) and BCF (Hz).

Two fluorochromes were combined to evaluate MI and AI: propidium iodide (PI), that stains cells with damaged plasma membrane, and fluorescein isothiocyanate-conjugated peanut (*Arachis hypogaea*) agglutinin (PNA-FITC), that stains damaged/reacted acrosomes. A total of 200 sperm cells were evaluated per sample by fluorescence microscopy (Nikon Eclipse E200, Nikon Instruments Inc., New York, USA): PI-negative cells were considered to preserve the MI and PNA-negative cells were considered to preserve the AI.

To assess MI by the EN staining technique, 5 µl drop of diluted sperm was mixed with 10 µl of the eosin-nigrosin solution. The HOST was performed by diluting 5 µl of sperm in 100 µl of hypotonic solution (100 mOsmol/kg). After 30 min at 37 °C the reaction was stopped by adding 100 µl of 2% glutaraldehyde solution and the percentage of sperm showing coiled tail was assessed using a phase contrast microscope (Axiostar plus, Carl Zeiss Microscopy GmbH, Jena, Germany). To assess AI in glutaraldehyde fixed samples, 5 µl sperm was diluted in 100 µl of 2% glutaraldehyde solution to calculate the percentage of normal apical ridge (NAR) using a phase contrast microscope. MI and AI were always evaluated in 200 cells per sample.

### Sperm freezability assessment

Sperm freezability, also known as sperm cryoresistance or freezing resistance, is the ability of sperm cells to withstand the freezing process. In this study sperm freezability was assessed by the comparison of post-thaw sperm parameters between conditions of study.

### Sperm incubation

Frozen-thawed sperm was gently centrifuged (500 × *g*, 5 min) in order to remove the freezing extender and then diluted to a concentration of 40 × 10^6^ sperm/mL in TALP medium. Each sample was divided into two aliquots using two different medium: TALP- (NCA condition) or TALP + supplemented with 5 mg/mL of bovine serum albumin (BSA) and 25 mM NaHCO_3_ (CA condition)^98,99^. Diluted sperm samples were incubated in the corresponding medium during 3 h at 39 °C in an incubator with 5% CO_2_ and humidified atmosphere.

### Evaluation of sperm PTP by western-blot

Protein extraction was performed using 4 × 10^6^ sperm cells that were washed in PBS (phosphate buffered saline) and resuspended in Laemmli sample buffer^100^ prior to being boiled for 5 min. Extracted proteins were loaded on 10% SDS-PAGE gels and run at 40 mA. Proteins were transferred to PVDF membranes (Millipore, CA, USA) at 250 mA (90 min). Membranes were blocked with 5% BSA in PBS with 1% Tween 20 (TPBS) for 1 h at room temperature (RT) and overnight at 4 °C. Membranes were incubated with anti-phosphotyrosine primary antibody for 1.5 h at RT (1:10000 in 1% BSA/TPBS; 4G10, Millipore, Madrid, Spain), washed with TPBS and incubated with peroxidase conjugated secondary antibody for 1 h at RT (1:10000 in 1% BSA/TPBS; 170-6516, Bio-Rad Laboratories, CA, USA). The Precision Plus Protein Dual Color Standards (Bio-Rad Laboratories, CA, USA) was loaded in the first lane of the gel as a molecular weight standard. Band visualization was performed with a developing solution containing 100 mM Tris, 0.009% H_2_O_2_, 250 µM Luminol and 40 µM Coumaric acid. Immunoblot images were captured using the Amersham Imager 600 (GE Healthcare UK Limited, UK) and the PTP signal was semiquantified with the ImageQuantTL 8.1 (GE Healthcare UK Limited, UK).

### Immunolocalization of sperm PTP by indirect immunofluorescence (IIF)

Sperm PTP was assessed by IIF as previously described^32^. Samples were fixed with 2% p-formaldehyde/PBS during 60 min at 4 °C and centrifuged (270 × *g*, 10 min). The pellet was resuspended in a blocking solution containing 4% BSA/PBS. Samples were blocked overnight at 4 °C, centrifuged (270 × *g*, 10 min) and the pellet was resuspended with PBS and smeared on slides (30 µl drop). Slides were washed 3 times with PBS and incubated with monoclonal anti-phosphotyrosine primary antibody (1:300 in 1% BSA/PBS; 4G10, Millipore, Madrid, Spain) during 1 h at 4 °C in a wet chamber. Slides were washed again before being incubated with the fluorescein-conjugated goat anti-mouse secondary antibody (1:400 in 1% BSA/PBS; Bio-Rad Laboratories, Madrid, Spain) during 1 h at 4 °C in a wet dark chamber. Control slides were incubated following the same procedure but replacing the primary antibody by 1% BSA/PBS solution. Finally, slides were mounted with Fluorescent Mounting Medium (Dako, Carpinteria, CA, USA) and coverslips (40 × 22 mm). The localization of sperm PTP was evaluated with a Leica DMR microscope equipped with bright filed and fluorescent optics (excitation 450–490 nm: B2-A filter, 4003). The images were captured using a microscope digital camera system (Zeis AxioCam HRc). Sperm cells were categorized in four subpopulations: sperm with no fluorescence (pattern I), sperm with ER fluorescence (pattern II), sperm with acrosome fluorescence (patterns III-IV) and sperm with tail fluorescence (patterns V-VIII). Two patterns were included in the acrosome fluorescence subpopulation: sperm with fluorescence only in the acrosome (pattern III) and sperm with fluorescence in the acrosome and ER (pattern IV). Additionally, two types of pattern IV were distinguished: sperm showing fluorescence in the apical part of the acrosome (pattern IV-a) and sperm showing fluorescence in the whole acrosome (pattern IV-b). Four patterns were included in the tail fluorescence subpopulation: sperm with fluorescence only in the tail (pattern V), in the tail and ER (pattern VI), in the tail and acrosome (pattern VII) and in the tail, acrosome and ER (pattern VIII). A total of 200 sperm cells per sample were evaluated to calculate the percentage of each subpopulation. Control slides showed no fluorescence as expected, confirming the antibody specificity (Supplementary Fig. 1).

### Study design

#### Experiment 1: Effect of sperm source (epididymal or ejaculated) on freezability (slow or ultrarapid)

Each ejaculate (n = 25) or epididymal sperm sample (n = 12) was divided into two aliquots and cryopreserved by slow-freezing or ultrarapid-freezing. Sperm quality parameters (motility, MI and AI) were assessed in fresh samples immediately after collection and after the thawing/warming process. Post-thaw parameters were compared between both sperm sources in order to compare epididymal and ejaculated sperm freezability. Post-thaw parameters were compared between both freezing techniques in order to evaluate the effectiveness of the freezing techniques.

Due to the limited equipment in the “field laboratory” during epididymal sample collection, fresh epididymal sperm motility was assessed subjectively, AI was assessed in glutaraldehyde fixed samples and MI was assessed by EN and by HOST. Fresh ejaculated sperm and post-thaw quality parameters of both epididymal and ejaculated samples were evaluated in the laboratory at the INIA research center (Madrid, Spain) where motility variables were assessed by CASA (SCA software) and AI and MI were assessed by fluorescence microscopy.

#### Experiment 2: Effect of sperm source (epididymal or ejaculated) on capacitation status

Sperm capacitation status was compared between epididymal and ejaculated samples using sperm cryopreserved by slow-freezing. Sperm PTP was assessed by western blot in ejaculated (n = 5) and epididymal (n = 4) frozen-thawed sperm samples in NCA and CA conditions at 1 h incubation. Localization of phosphorylated proteins was assessed by IIF at 0, 1, 2 and 3 h incubation (n = 5) and motility variables were also assessed at 0, 1, 2 and 3 h incubation (n = 5) using CASA system (ISAS software). The kinetic parameters VCL, LIN and ALH were used for cluster analysis since these parameters have been described as good classifiers for sperm clustering in domestic ram^86,101^.

### Statistical analysis

The effect of sperm source on sperm freezability (Exp. 1) and the effect of sperm source and incubation media on immunoblot results (Exp. 2) were analyzed by the t-test using the SAS software (2016 version, SAS Institute Inc., Cary, USA). The effect of sperm source, incubation medium and time on the PTP immunolocalization (Exp. 2) were analyzed by repeated measures ANOVA using the SAS software.

Sperm cells were grouped in three clusters using IBM SPSS v.19 (SPSS Inc. Chicago, IL, USA) by a non-hierarchical *k*-means clustering analysis defined by VCL, LIN and ALH. A total of 4850 motile sperm cells were included in the analysis. Clusters were analyzed by a multiple mixed effects logistic model using Stata v.15.1 (Solingen, Germany) to estimate and test the probability to belong to cluster 1, cluster 2 or cluster 3, considering sperm source, medium and time as fixed effects and the variability between individuals as random effect. Differences between the percentages of sperm belonging to each group (named contrasts) are analyzed with this model. When significant differences were found pairwise multiple comparisons was performed by Fisher´s protected least significant difference test. Significant differences were considered when p < 0.05. Results are expressed as mean ± standard error of the mean (s.e.m.).

## Supplementary information

Supplementary Figures

## Data Availability

The data sets generated during the current study are available from the corresponding author upon reasonable request.
