# Supplementary figures and images for: Epididymal and ejaculated sperm differ on their response to the cryopreservation and capacitation processes in mouflon (Ovis musimon)
Source: Sci Rep. 2019 Oct 30;9:15659. doi: 10.1038/s41598-019-52057-0 (PMC6821854; doi:10.1038/s41598-019-52057-0)

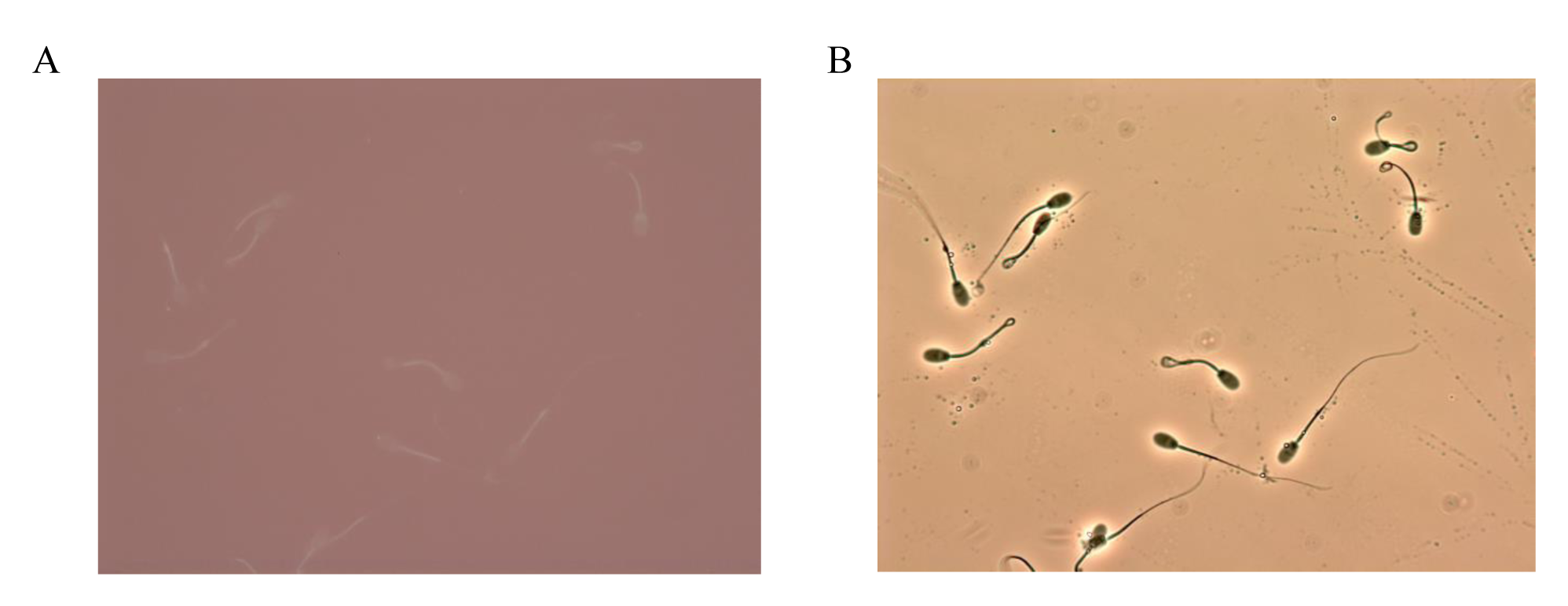

Supplement: Supplementary file 1 — Supplementary Figures [file 41598_2019_52057_MOESM1_ESM.tif]
